# Rest: A Health-Related Phenomenon and Concept in Caring Science

**DOI:** 10.1177/2333393615583663

**Published:** 2015-04-29

**Authors:** Margareta Asp

**Affiliations:** 1 Mälardalen University, Eskilstuna, Sweden

**Keywords:** caring, concept development, Gadamer, health and well-being, hermeneutics, lived experiences, Merleau-Ponty, nursing, phenomenology

## Abstract

Rest is a health-related phenomenon. Researchers have explored the phenomenon of rest, but further concept development is recommended. The aim of my study was to develop and describe a concept of rest, from interviews with a total of 63 participants about their lived experiences of rest. I performed the developing process in two stages: first with descriptive phenomenology and second with a hermeneutic approach. The concept of rest is comprised of the essences of both rest and “non-rest,” and there is a current movement between these two conditions in peoples’ lives. The essence of rest is being in harmony in motivation, feeling, and action. The essence of non-rest is being in disharmony in motivation, feeling, and action. The essences reveal some meaning constituents. Health care professionals and researchers can use the concept as a frame of reference in health care praxis and in applied research.

People need rest to recover from strains and illness to maintain health (Han, 2013; Svenson, 1990). Although rest has been regarded as a fundamental phenomenon for human recovery, few researchers have studied the meaning of rest in a deeper sense. People usually take rest for granted in their ordinary lives and the phenomenon of rest has hardly been reflected on or analyzed in a scientific way. Scientific work includes concepts and if a concept is poorly described it can be developed from qualitative studies (Morse, 2004) and from people’s lived experiences of the phenomenon (Asp & Fagerberg, 2005). Concepts function as tools, help us to be aware of phenomena in praxis and to integrate levels of science and reality (Eriksson, 2010). I define concepts according to Rodgers’s (2000) definition:

Concepts are cognitive in nature and are comprised of attributes abstracted from reality, expressed in some form and utilized for some common purpose. Consequently, concepts are more than words or mental images alone. In addition, an emphasis on use alone is not sufficient to capture the complex nature of a concept. (p. 33)

Health care professionals can use concepts as frame of references in health care and researchers as a theoretical perspective in applied research.

## Adjoining Concepts to Rest

Rest has similarities with comfort. Kolcaba (1994) developed the concept comfort by a semantic analysis and has developed the concept into a theory (Kolcaba, 2003). The comfort concept comprises the aspects ease, relief, and transcendence, a condition when humans are lifted from problems and pain. Kolcaba (1994) combined these three aspects with physical, psycho-spiritual, social, and environmental aspects in a taxonomic structure with 12 cells. The structure is supposed to represent patients’ needs for comfort in a holistic perspective. The concept’s relation to health is evident, but it is difficult to separate and order experiences of comfort in relation to the specific dimensions that represent the human being.

McIlveen and Morse (1995) described the role of comfort in the history of nursing care. Comfort was a central goal for nursing and medicine at the beginning of the 19th century. In the later part of the century, comfort was a minor aspect of nursing and partly replaced by medical treatments. The researchers (McIlveen & Morse, 1995) describe comfort as nursing strategies to bring the desired rest for the patient and rest plays an important role in the recovery process. The theory of comfort is patient centered but is mainly focused on nursing comfort strategies that give relief, satisfaction, confidence, and calm, aspects that belong to the concept comfort (Morse, 1997, 1998). Morse developed the theory about comfort from empirical studies and recommends that researchers study relationships between comfort and other words associated to recovery. Rest is such a word.

When researchers develop concepts and theories, the assumptions they make on ontological and epistemological levels must be congruent with their statements on methodological levels (Kirkevold, 2001) and theoretically linked (Morse, 2004). In this study, the concept of rest is linked to the concepts of health and the human being, which are paradigmatic concepts in caring science and nursing.

## Ontological and Epistemological Foundation for Concept Development in Caring Science

Caring science has a patient perspective, researchers develop concepts and theories from patients’ experiences of health, suffering, and care, and nurses as well as other health care professionals can use these theories and concepts in praxis (Dahlberg & Segesten, 2010; Dahlberg, Todres, & Galvin, 2009).

In this study, I regard health as movements in a human’s life processes (Dahlberg et al., 2009; Eriksson, 1995). Eriksson (1995) described health in the dimensions “to have health,” “to be health,” and “to become health.” These three dimensions are complementary. To have health means to have a healthy behavior. It is an objective aspect of health. In this dimension, health promotes by recommendations and prevention of diseases. To be health means humans strive to achieve balance in life and to satisfy their human needs. Health, in this dimension, has both objective and subjective attributes. To become health means that humans experience integration in a deeper sense. The dimension concerns humans’ innermost desire for life, love, and meaning. To strive for health in this dimension implies to have focus on the motives, the driving force behind humans’ action, to be aware of one’s responsibility as a human being and in relation to others.

The human being is regarded as a multidimensional unit of body, mind, and spirit (Dahlberg & Segesten, 2010; Eriksson, 2002). Humans constantly influence and are influenced by the environment. These assumptions are congruent with a lifeworld perspective with commonalities found in Husserl’s, Merleau-Ponty’s, Heidegger’s, and Gadamer’s philosophies (Dahlberg, Dahlberg, & Nyström, 2008). The lifeworld perspective comprises the concepts lifeworld, lived body, and intentionality.

The lifeworld is the world in which we live our daily lives, in an ordinary, natural attitude. The lifeworld is both immanent and transcendent, which means that the idea of lifeworld bridges the gap between the human’s inner and outer world. Accordingly, we can regard a concept both as a mental image and as related to a phenomenon at the same time (Asp & Fagerberg, 2005). Merleau-Ponty (1962) described the human being undergoing an experience as a lived body. He regarded the lived body as an indivisible, ambiguous existence of the subject-object and consequently not just the sum of its own parts. The body acts as a form of a whole and is perceived as a whole. Intentionality is a theory about how we perceive the world. According to that theory our consciousness is always directed toward something, so the intentionality comprises of both the conscious act and the phenomenon. This theory confirms the idea that there are relationships between a sign, a meaning/concept, and a phenomenon (Asp & Fagerberg, 2005; Ogden & Richards, 1923). These assumptions are a foundation for the concept development of rest.

The term *rest* is both a noun and a verb. Rest means “respite from labor or exertion of any kind” and “refreshment or repose obtained by a pause in activity” and “freedom from trouble, distress, molestation, aggression and so forth; a state of peace” (Oxford English Dictionary, 2012). Westrin (1909) defined rest as a relative phenomenon and always related to movements and Swedenberg (2000) described rest as a process overcoming physical or mental fatigue or weariness. Accordingly, the term *rest* comprises of both a process (to rest) and a condition (being in rest).

## Research About the Phenomenon of Rest

Allison (1970) performed an explorative study about the phenomenon of rest described by healthy men in daily living. She derived two general conclusions from the findings. First, the phenomenon of rest varied with the individual and could involve any kind of activity. Second, rest involved a change from a previous state. The rest activity state was dependent on the previous state. The researcher identified four attributes in association with rest: the notion of freedom from mental stress and relaxation, freedom to do what is liked and relaxation, pleasure in performance and/or accomplishment, and the notion of restoration.

Mornhinweg and Voignier (1996) found that nursing teachers perceived patients’ rest as inactivity, relaxation, and sleep. But the nursing teachers themselves perceived rest as anything that gave them freedom from daily endeavor, leisure, and recovery to the entire human being: to body, soul, and spirit. The results show that the paradigm from medicine has influenced how nursing teachers perceived patients’ needs, when the physical body was mainly in focus for care.

Occupational therapist researchers have explored the phenomenon of rest by studying how rest is described in literature from different religions and through an explorative qualitative study (Nurit & Michal, 2003). The results show that rest means an activity that is personal, quiet, and effortless, and it is possible to experience rest alone or together with others.

Researchers have mainly studied rest in explorative studies and they (Mornhinweg & Voignier, 1996; Nurit & Michal, 2003) recommend more studies for concept development from peoples’ experiences of rest. According to Morse (2004), qualitative researchers must develop concepts and theories to be more than narrative descriptions to be useful for praxis and research. The aim of the present study was to describe the concept of rest, developed from people’s lived experiences of the phenomenon, with a caring science perspective based on lifeworld theory. Health care professionals and researchers will have the opportunity to use the concept as a frame of interpretation in health care and in applied research.

## Method

I used a model (Asp & Fagerberg, 2005) for concept development based on a lifeworld perspective and on Merleau-Ponty’s (1964) philosophy of language, which has phenomenological, semilogical, and pragmatic dimensions. According to the model, when a concept is poorly developed, the developing process goes from phenomenon to concept, which I performed in two stages.

### Stage I

In the first stage, I performed a descriptive phenomenological study (Asp, 2002) based on descriptions of rest from 19 participants (No. 1–19), selected according to a principle of variations in experiencing the phenomenon (Dahlberg et al., 2008). To get a general description of rest from the analysis, the sample varied regarding sex (11 women, eight men), age (between 18 and 85 years), occupations (white-collar, blue-collar, and unemployed), and health situation (one participant with a disability, 10 patients with different diseases cared for on medical and surgical wards, and eight participants in good health). The first seven interviews influenced how I selected the next participants. For example, the participants described several kinds of activities as restful, and therefore I selected a participant with severe functional disabilities with action limitations to be interviewed next. This is an example of how the researcher can be open-minded to the phenomenon in the data collection and can try to be aware of the unexpected (Giorgi, 1997).

I transcribed the interviews and analyzed them according to a descriptive phenomenological approach (Dahlberg et al., 2008; Giorgi, 1997), which according to Giorgi (1992) is a relevant research approach for basic research. The result is a description of the phenomenon of rest, its essence and meaning constituents (Asp, 2002). The assumed connections between the phenomenon of rest and the mental image of rest, implies that the essence and the meaning constituents represent the mental image of the concept rest.

The essence comprises of the aspects that are invariant in the participant’s descriptions of the phenomenon and an essence can be compared with what Morse (2004) described as the characteristics that must always be present in a description for it to belong to a particular concept. The meaning constituents are variations of the concept and described on a less abstract level than the essence. With a descriptive phenomenological analysis, it is possible to develop a concept with attributes on different levels of abstraction. The researcher must determine the level of abstraction for the concept, according to how it is supposed to be used. In this study, I describe the essence and the meaning constituents on a scientific level of abstraction to be useful as a frame of reference in praxis and in applied research. The results from the descriptive phenomenological study were a point of departure for further concept development in the next stage.

### Stage II

In the second stage, I developed the concept of rest further by analyzing people’s descriptions of their lived experiences of rest to refine and validate the concept. I purposively selected 44 participants (No. 20–63) for interviews to get variations of descriptions of rest. The participants’ ages varied from 16 to 82 years and included 20 men and 23 women, with different professions. The interview length varied between 20 to 40 minutes and focused on the participants’ experiences of rest and restful conditions, and began with the questions: What does rest mean to you? How do you rest? Can you tell me about a restful experience? The interviews continued with follow-up questions to deepen and specify the participants’ descriptions. The interviews were recorded and transcribed verbatim.

I performed the analysis influenced by how Gadamer described the act of interpretation on an epistemological level. According to Gadamer (1998), every interpretation begins with a prejudice that confirms, contradicts, and reorganizes in interplay with the “things.” In this analysis, the description of rest, its essence and meaning constituents from the previous study (Asp, 2002), was regarded as one aspect of my prejudice in the act of interpretation. The “things” were the transcribed interviews in Stage II. The process of interpretation comprises of three acts: understanding, explanation, and application (Gadamer, 1998). I compare the act of understanding, with the intention to suspend prejudice and with openness try to put the prejudice in play. In this study, I performed the understanding act by analyzing data with suspended pre-understanding of previous descriptions about the phenomenon, with an intention to recognize something unexpected. In each interview, I identified and reflected on meaning units of rest in relation to the meanings of the entire interview to get a relevant transformation of meanings of rest. The transformed meanings were ordered in clusters (Dahlberg et al., 2008).

In the act of explanation, the interpreter reflects on what has emerged in the act of understanding. I reflected on the clusters in relation to the description of rest from the previous study (Asp, 2002) and a revised, nuanced understanding of the concept emerged, which is presented in the “Results” section. The application act (Gadamer, 1998) is how general knowledge can be understood and applied in a new way in a specific situation. I describe examples of the application act in the “Discussion” section.

### Ethical Considerations

The research ethic committee at Örebro County Counsil in Sweden gave the approval for the study in Stage I (Asp, 2002; Diary no. 500:16 190 00). The study in Stage II did not include patients and according to Swedish ethical legislation, an approval for research is not necessary in that case. The participants received both written and oral information about the study and were told that they could withdraw from the project at any time and they were guaranteed confidentiality.

## Results

The result from the analysis in Stage II is a description of the concept of rest. The essence and the meaning constituents are a mental image of the concept, which have connections to the phenomenon. I describe the concept of rest and use the word “one” instead of “the participant” so that readers can understand the phenomenon from their own perspectives.

The concept of rest comprises its duality—“non-rest.” It is a movement, a rhythm between these two human conditions. The essence of rest is an experience of harmony concerning one’s feelings, actions, and motivation. This implies that there is a capacity for actions, which is carried out in accordance with a sensation of pleasure. Rest appears when one’s needs and longing correspond to the shape and character of the environment. Rest takes many different states from calm, demand-free, and peaceful conditions to conditions where one is open and perceptive to pleasurable impressions. The essence of rest is characterized by a sense of confidence and trust in one’s own inviolable human dignity and in being loved.

The essence of non-rest is a disharmony in motivation, feelings, and action. This implies that there is a division, both in one’s will and capacity for action. It can also mean a conflict between feelings and the will to take action, as well as a division between one’s will and ability to feel. These conditions drain the person of energy and become more obvious the longer the period of non-rest continues. The all-embracing rhythm and progressive movement between the dual conditions of rest and non-rest is driven by human’s motivation to take responsibility and to search for meaning, in every new moment of life. In rest, there is a prerequisite for movement toward non-rest. This means that one becomes more balanced and obtains beneficial energy, before one moves again toward a state of non-rest. In the state of non-rest, there are conditions for a movement toward rest. This implies that one becomes aware of sensations of tiredness or weariness; one selects and performs states of restful activities according to one’s will and feelings.

The essential structure of rest and non-rest appears in some meaning constituents: being in a rest rhythm, letting go in confidence, being accepted without judgment, dwelling in calm and peace, perceiving pleasurable sensations, strained between limited resources and demanding expectations, and perceiving disinclination and/or ennui.

### Being in a Rest Rhythm

Being in a rest rhythm implies that one experiences a rhythm between the states of non-rest and rest. The rest rhythm is visible in the cultures and societies as weeks, where the weekend implies rest. The daily and seasonal rhythms in nature are definite and one can live one’s life in accordance to these rhythms and have strong support to experience rest in a speeded up existence: “When I am out in nature, I enter another mental state and then other values apply and I abide by them and also want to do it . . . experience this rhythm of nature, this breathing space.”

The rest rhythm also implies to take brakes during the day and to temporarily change one’s attention from non-restful activities toward restful activities to recover. “To regain something that you can live on for a while.” When mentally demanding activities have been performed for a while and the experiences of non-rest are evident, there is a need to switch on to a mentally restful activity, which could be a physical activity. “You clear your mind when you are out running/It is a form of rest.”

### Letting Go in Confidence

Being human implies living in responsibility, which can sometimes be demanding, “you need to be responsible, you need to grow up.” Rest means that one can temporarily allow oneself to let the responsibility go and experience harmony concerning one’s feeling, actions, and motivation. “You leave all the demands you carry around and things you should keep up with and just rest up for a while in order to cope later.” Rest means to experience harmony and having confidence in others, like patients can trust health care professionals and feel secure that they act according to what is best for the patients.

Confidence in God or some higher power is experienced as restful, “a foundation I constantly rest on.” Being confident implies having a strong belief in one’s inviolable human value and in being loved. “It means security in knowing that somewhere deep down, I am loved, valuable, this inviolable dignity that no one can take away from me. This is a deep rest.” The human value is perceived to be based on the fact that one exists and not on one’s actions. This profound, ontological aspect of rest is not influenced by the movement between restful and non-restful activities. However, a prolonged state of non-rest implies that one can become exhausted and this condition can affect one’s self-confidence.

### Being Accepted Without Judgment

Being accepted without judgment is an aspect of rest that can be experienced in relationships where one feels safe and experiences tolerance from others, without feeling them to be too obtrusive. There is no need to behave in an expected manner and it is possible to relax and recover, implying experiences of harmony concerning one’s feelings, action, and motivation. In a restful relationship, one can get confirmation and reflect on one’s thoughts, feelings, and actions. For a patient, it is especially important to be accepted and confirmed by health care professionals:

This particular kindness. They take part of course in our lives. They say nothing as they nod and give a smile. You suck everything in when you’re at a place like this: You are more dependent on it when you are not really healthy yourself.

A restful relationship can involve activities based on common interests and means that people voluntarily participate in actions together and accomplish something with another person with a feeling of pleasure and harmony. For example, singing in a choir, “The feeling that your voice is doing something with others, when you sing, and create a whole out of four meetings, with other people. You get goose bumps.” The relationship between the patients and the health care professionals can be restful and the common interest is the patients’ recovery.

### Dwelling in Calm and Peace

To rest by taking the time to dwell and slacken one’s pace or even ceasing activities for a while implies renewing one’s energy and strength when one is tired. It also means to temporarily detach from pressure and demands and take time to relax and recover. It is time to reflect on earlier experiences and become more integrated and whole as a person. “In silence you meet yourself and it can be hard, but it is necessary.” One aspect to dwell on is to slacken one’s body and screen off all external impressions to rest. Another aspect is to experience calm and peace while exposing oneself to a small amount of outside impressions. In a condition of dwelling, the need for relaxation is in focus and the kind of activity is of minor importance. “When I go into town and I just go and look spontaneously, so you can sit and have a coffee watch people go by. I think that’s rest.” There is a distance, not just in physical form, from the situations or activities that are non-restful and this gives opportunities to master experiences of uncertainty and worry. The environment is restful when it has a cheerful atmosphere, comprising “calm, ordinary traces of human life and consideration,” a place where one can dwell and experience harmony concerning one’s feeling, action, and motivation.

### Perceiving Pleasurable Sensations

Perceiving pleasurable sensations is a form of rest, experienced when one is open to impressions and activities that give energy and joy. This type of activity is of major importance and specific for the actual situation. There is an agreement between being motivated and being able to take part in this activity, without pressure and without a feeling of being constrained by time. This implies that one derives pleasure and vigor from the situation, which enables seeing the life situation from a distant or new perspective. This form of rest gives temporary freedom from compulsion and weariness.

It is possible to let go when focus is on the pleasure-filled activity. This involves stimulating impressions, as well as atmospheres of beauty. Experiences of beauty mean an intensive openness and presence and at the same time absence from the situation. The sensations can be involved in the beauty of nature as well as in music and art: “It was so wonderful. Then I felt harmony. These are the moments that are left when everything else is screened out. There was some sort of quiet that came.” By being open, one can consume sensations of pleasure, experienced as rest. “All sensations, it may well be that it smells good on some flowers, that is rest or listening to the blackbird singing / waves lapping. It is also a kind of mental rest.” It is possible to experience restful pleasure by performing creative activities, when one feels weary and compulsion:

Rest I can feel when I’m doing creative things that I like. I think that’s when I feel comfortable with a particular situation where I feel calm and peaceful as I relax . . . I find peace of mind when I am doing something that I find interesting and stimulating.

The creative activity is restful if it is in harmony with one’s motivation and feelings, filled with pleasure at that moment.

### Strained Between Limited Resources and Demanding Expectations

The essence of non-rest constitutes being strained between one’s limited resources and demanding expectations, which implies experiences of disharmony. There is a conflict between too high personal expectations for action related to personal prerequisites and resources. “You can put too high demands on yourself and then everything goes crazy.” The demands can also come from external sources in one’s life situation. The following quotation elucidates a state of non-rest for an older spouse who cared for her husband with disabilities:

He was supposed to get help with everything, he can’t do anything, I must help with everything I am so tired, to the point of exhaustion / He went to bed quite early, but he did not sleep. He was asking for tablets, tablets. But I could not give him all the tablets he wanted.

She felt strained when she could not satisfy his demands.

Patients can experience non-rest in unfamiliar clinical settings when they have limited resources in a weak body related to demands from health care professionals. “As a patient you feel a little oh no, am I in the way, or do the staff think it is difficult? Why would she come here and interfere?” Non-rest also appears when there are too many activities to perform within a limited time and feelings of disharmony emerge. Control is lost, the ability to see the whole situation is lost and one feels split and strained: “It is really difficult to find the time for everything with work, home, training, food and all the people around who make demands.”

If one continues with an activity for a long time without breaks for rest, one becomes drained of bodily and mental resources. This can occur, for example, in services or care situations with demanding relations to others. This implies that one can temporarily lose the ability to be aware of others’ feelings and meet their needs:

It wears you down when you meet so many people. You have to give yourself and be present. And I am not made of stone. It affects me when I meet people in grief. If I can’t keep my cool and feel empathy, which I must feel, then I can’t help anybody / you become a blunt knife.

By listening to the bodily signals, one can be aware of when it is time for restful activities and move the attention from non-restful activities to states of rest, from experiences of disharmony to harmony, concerning one’s feeling, action, and motivation.

### Perceiving Disinclination and/or Ennui

A constituent of non-rest is perceiving disinclination in action. These are situations when one performs activities that one must and is able to perform, but does not like and gets feelings of weariness and ennui. There is a disharmony concerning one’s feeling, action, and motivation. The following quotation elucidates this constituent in a situation when a family father gets compulsions from the rest of the family about performing activities that he dislikes during the vacation. “You have to meet all these expectations from the rest of the family and then it becomes a compulsion to do so and I cannot rest.” Being obliged to take care of a relative, without feeling motivated is experienced as disinclination:

Being forced to, that is something terrifying. That was how I felt when my father moved in. I was trapped in a cage. I was trapped in school until 4pm and then I was trapped at home by a father and a dog. And that was when I felt that anxiety. Would my life just continue in this way? Anxiety, anxiety. Duty here, duty there.

The activities are not in harmony with one’s feelings and motivation, which implies that one becomes drained of energy and needs rest.

## Discussion

The concept of rest comprises movements between the essence of rest and non-rest. The result is in line with the definition of rest, as a relative condition and always related to movements (Westrin, 1909) and, according to Alison (1970), that rest involves a change from a previous state. This confirms the present result that both rest and non-rest belong to the concept rest. There are two dual conditions: harmony respective disharmony concerning one’s feeling, action, and motivation that constitute these conditions. The essences are on a level of abstraction that Morse (2004) labeled as high and the meaning constituents can be placed on a mid-level, with a lower abstraction.

### The Concept of Rest Related to the Ontological Assumptions

The concept of rest has been developed from peoples’ descriptions of their lived experiences of rest. When taking a natural attitude, the participants do not talk about the phenomenon “out there” or within “themselves” separately, instead the immanent and the transcendent aspects of rest are intertwined in the descriptions. Accordingly, each meaning constituent comprises both aspects in the lived body and the lifeworld. The concept enables reflections about rest to comprise of the whole human being without separating and analyzing physical, psychological, existential, or social aspects of rest, as in the concept of comfort (Kolcaba, 2003). The results show that experiences of rest imply a sense of harmony in the lived body.

“Being in a rest rhythm” can be related to the meaning of health as a process (Eriksson, 1995) and as a movement in life processes (Dahlberg et al., 2009). The living body is never completely calm and in stillness while resting. Although the body may at times be inactive, the mind is still active during rest. The health concept signifies the meaning of integration, leading to health (Eriksson, 1995). Health is always changeable and a person’s life processes are always moving between a state of disintegration and integration, a process of becoming and the concept captures the human’s most inner desire for life, meaning and striving for integration. This is in line with the concept of rest. The circularity between conditions of rest and non-rest characterizes human life and searching for meaning.

### The Concept of Rest Related to Adjoining Concepts

The rest concept has similarities with the concepts of comfort. The meaning to dwell can be compared with “ease” in the comfort theory, pleasure-filled sensations give similar experiences as described by “transcendence” and all meaning constituents in the concept of rest involve “relief” (cf. Kolcaba, 1994). The concept rest has similarities with aspects in Morse’s (1997) comfort theory with nursing strategies to ease, relieve, and to make strong. The differences in the concept of rest compared with the concepts of comfort are that the comfort theory mainly focuses on nursing strategies, whereas the concept of rest focuses on meanings of rest from a patient perspective. The concept of rest is developed from a lifeworld perspective, implying that the meaning constituents are formulated from the participants’ perspectives. The concept rest also comprises non-rest and rhythm: the movements between rest and non-rest. The concept elucidates that activities meant to be restful must fit the individual’s specific condition and the same activity is not necessarily restful for the individual under other conditions.

### Application

The concept’s mental image comprises variations of rest with surplus meanings. Concepts are tools to link theory to practice (Eriksson, 2010; Morse, 2004). The concept can be used by health care professionals in their praxis as a frame of interpretation to be aware of and reflect on patients’ prerequisites and need for rest. The concept enables health care professionals to communicate about the phenomenon in their professional language. The variations of rest are put into words that help the health care professionals to focus on the patient’s lifeworld and the level of abstraction implies that the patients must participate in their care process to confirm and decide if the restful activity is in harmony with their motivations and feelings. This can withdraw to a patient-centered care (Dahlberg et al., 2009). Based on the concept of rest, the health care professionals have different rest interventions to suggest to the patients. The health care professional can reflect if the patient has a rest rhythm in life and how to arrange the environment to give the patients prerequisites for rest rhythms. What kind of caring/nursing interventions does the patient need to experience rest as harmony concerning feeling, action, and motivation? Does he or she need to dwell in calm and peace or experience pleasurable stimulations? Is the atmosphere a kind that makes people experience that they are accepted without judgment? Is the patient in a condition of non-rest and strained between limited resources and demanding expectations? Or does the patient perceive disinclination or ennui, implying disharmony? By reflecting on this mental image of rest in connection to the patients’ situations, health care professionals and patients can interpret the patients’ needs for rest, applied in the specific situation. In an act of application, the general knowledge, here it is the concept of rest, can be understood and applied in a new way in a specific situation (cf. Gadamer, 1998).

In a high goal achievement society (Han, 2013), the concept of rest can elucidate what kind of prerequisites we need to experience rest and promote health, in the dimension to have health (Eriksson, 1995). For example, the concept elucidates health-promoting strategies for people to live in a rest rhythm in their ordinary lives, to dwell in the natural world, and to experience beauty in music and art.

Researchers can use the variations of rest in applied research as a basis for intervention studies to improve knowledge about restful strategies for people. Such research could also refine the concept of rest because application is a part of the concept development (Asp & Fagerberg, 2005). Furthermore, research is needed to develop a theory of rest on a middle range level.

### Methodological Considerations

The concept development of rest is based on empirical studies. In the present results, the meaning constituents are linked to data, which is a quality criterion for concept development (Morse, 2004). A second criterion is that the concept must be abstract enough to be useful in other contexts. I have transformed the meaning constituents to an abstraction level that could be useful for application in health care and health promotion.

The study in Stage II is a further concept development from the study in Stage I (Asp, 2002). In the analysis in Stage II, I have decreased the numbers of meaning constituents from 11 to seven. All variations of rest are still in the description of the concept, but I have synthesized some meaning constituents because of further abstraction. Few meaning constituents will make the concept more useful in praxis. When I performed the study in Stage II to develop the concept further, this implied challenges when I was supposed to be open to and be aware of new meanings in the data. The trustworthiness could be questioned if I used the previous results in a deductive way in the analysis. However, in the analysis in Stage II, I perceived new meanings of rest, for example, the participants’ experiences of high responsibility in life as a burden and a life sped up with a lot of activities that limited their time for rest.

**Figure 1.**
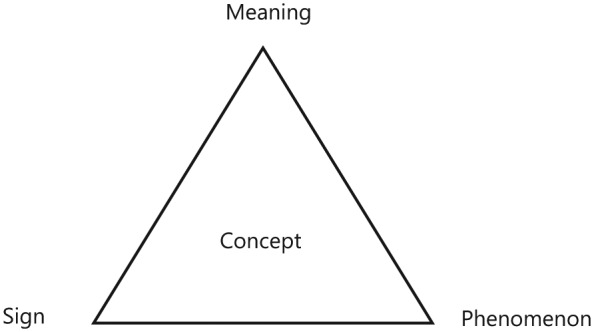
Aspects of a concept.

A concept comprises three aspects: sign, meaning, and phenomenon (Figure 1; Asp & Fagerberg, 2005; Ogden & Richards, 1923). I began this concept development process by using the sign “rest” in interviews with the participants to get their descriptions of the phenomenon. I analyzed the descriptions regarding the essence and meaning constituents of the phenomenon of rest. The result is the meaning of the concept.

### Conclusions and Further Research

The concept of rest that I have developed from peoples’ lived experiences is multidimensional and comprises the human being in the lifeworld. The concept comprises both rest and non-rest and the movement between these dual conditions. The meaning constituents of rest and non-rest are mental images that can be used to reflect on and become aware of peoples’ need for rest in their specific life situation and as a concept used in the care process, when the concept is related to the concept of health and health processes. My intentions for further studies are to clarify the concept of rest in relation to the concepts of fatigue, stress, comfort, and recovery. Another focus of my research is to implement interventions regarding variations of rest in health care to study their impact on peoples’ health.
